# From Seed to Spread: Lacrimal Sac Squamous Cell Carcinoma Blossoming Into Orbital Chaos

**DOI:** 10.7759/cureus.63452

**Published:** 2024-06-29

**Authors:** Chia Yaw Teoh, Kavitha Saravanamuthu, Wan Mariny W Md Kasim

**Affiliations:** 1 Ophthalmology, Hospital Serdang, Kajang, MYS

**Keywords:** neoplasm recurrence, surgical enucleation, endoscopic biopsy, lacrimal sac tumour, primary orbital squamous cell carcinoma

## Abstract

Non-cutaneous squamous cell carcinoma (ncSCC) is a rare malignancy, especially involving the orbital and lacrimal apparatus. Hereby, we present a case of recurrence of squamous cell carcinoma (SCC) in the left orbit after excision of lacrimal sac SCC and radiotherapy. She presented with acute painful visual impairment with a frontal headache, with imaging showing medial extraconal and intraconal mass. After confirmation of SCC from the biopsy, modified enucleation was done. However, the patient had a recurrence of SCC, and further debulking was performed with palliative measures. Noteworthy, ncSCC is a rare malignancy with an aggressive nature. Orbital SCC has the worst prognosis compared to conjunctiva or lacrimal sac SCC. Surgery remained the mainstay for higher survival, but chemotherapy and radiotherapy were not associated with a better prognosis, yet there is a lack of data regarding recurrence and its management. In conclusion, ncSCC is a rare and challenging disease that requires timely intervention with multiple disciplinary care, especially when it is spread from the lacrimal sac to the orbital.

## Introduction

Primary lacrimal sac and orbital squamous cell carcinoma (SCC) are rare malignancies compared to their counterparts. Among squamous cell carcinomas in ophthalmology, non-cutaneous primary sites were less common than cutaneous sites. Non-cutaneous squamous cell carcinoma (ncSCC) incidence was reported to be 0.68 per million populations, with the commonest site in the conjunctiva, followed by orbit, lacrimal apparatus, and mixed [1]. Lacrimal sac SCC (lsSCC) is common among epithelial lacrimal sac malignancies [2]. A 44-year epidemiologic study showed possible associations with male, elderly, and Caucasian individuals. One report showed the association of human papillomavirus (HPV) with lacrimal sac SCC [3]. Also, orbital and lacrimal apparatus involvement tend to undergo radiotherapy more than others, albeit not associated with a better prognosis. With detailed survival analysis, it was shown that those with risk factors were associated with low survival rates, while surgical intervention improved the survival of the patient [1]. Notably, lsSCC was mentioned to have a higher tendency of recurrence rate as compared to other subtypes of ncSCC. Here, we present a case of recurrent lsSCC as orbital SCC and challenges in management.

## Case presentation

**Figure 1:**
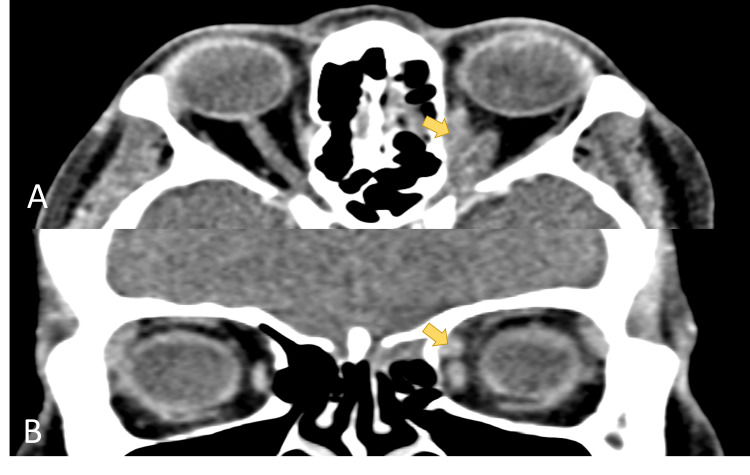
Computed tomography of the orbit during the first presentation. Axial view (A) and coronal view (B) of computed tomography of the orbit showed intraconal lesion (arrow) that extend superomedial and posteriorly involving the medial and superior rectus.

**Figure 2:**
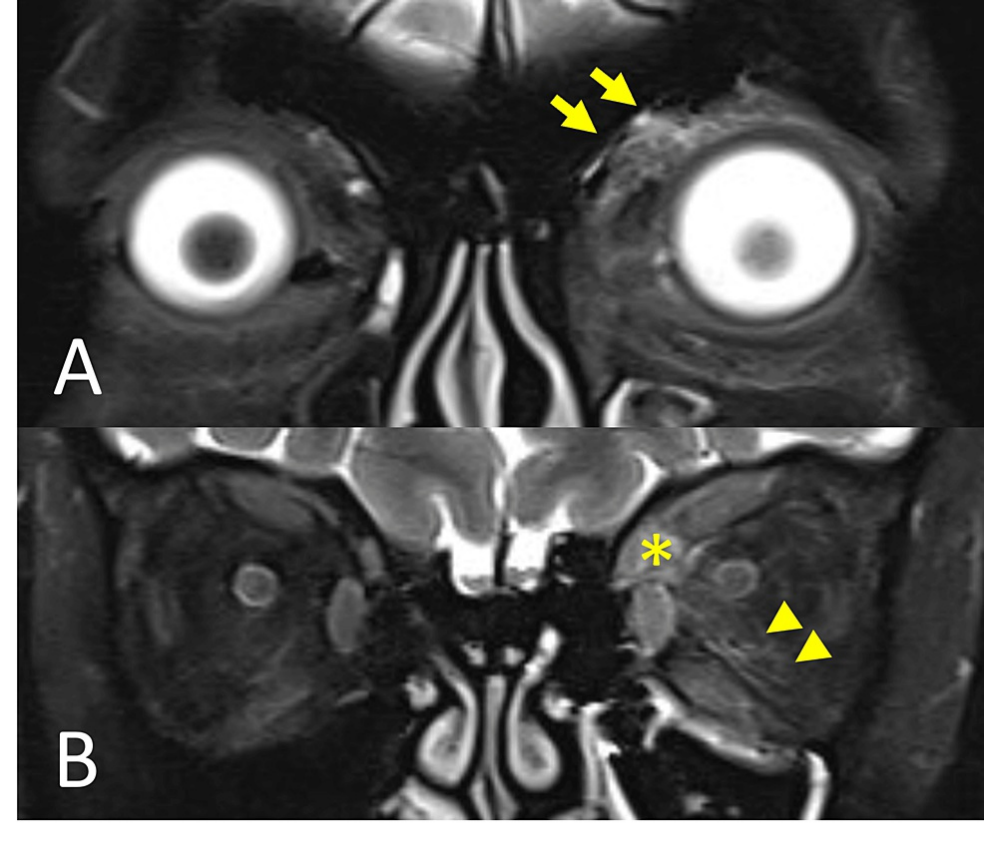
Coronal MRI orbit showed enhancing lesion over the left orbit. (A) Anterior orbital cut showed an enhancing ill-defined soft tissue lesion (arrow) over the extraconal superomedial periorbita compartment likely from the posterior orbit. (B) Posterior orbital cut showed an enhancing soft tissue lesion (asterisk) that occupied the superomedial compartment of the orbit while involved the superior rectus and medial rectus. Posterior two-thirds intraconal segment of the left optic nerve was surrounded by the extension of the lesion (arrowhead).

A 52-year-old lady with underlying hypertension and eczema with a history of lacrimal sac poorly differentiated SCC underwent excision and completed radiotherapy, presented with acute painful diminution of left eye vision associated with intensive frontal headache. The examination noted the vision of counting fingers and a positive relative afferent pupillary defect without proptosis or ocular misalignment. Contrasted computed tomography showed enhancing mass at the intraconal surrounding optic nerve, medial to superior rectus, and superior to medial rectus, as shown in Figure 1, which is further seen in magnetic resonance imaging of Figure 2. Positron emission tomography revealed fludeoxyglucose-18 uptake at the posteromedial aspect of the extra-orbital region, with no distant metastasis seen. 

**Figure 3:**
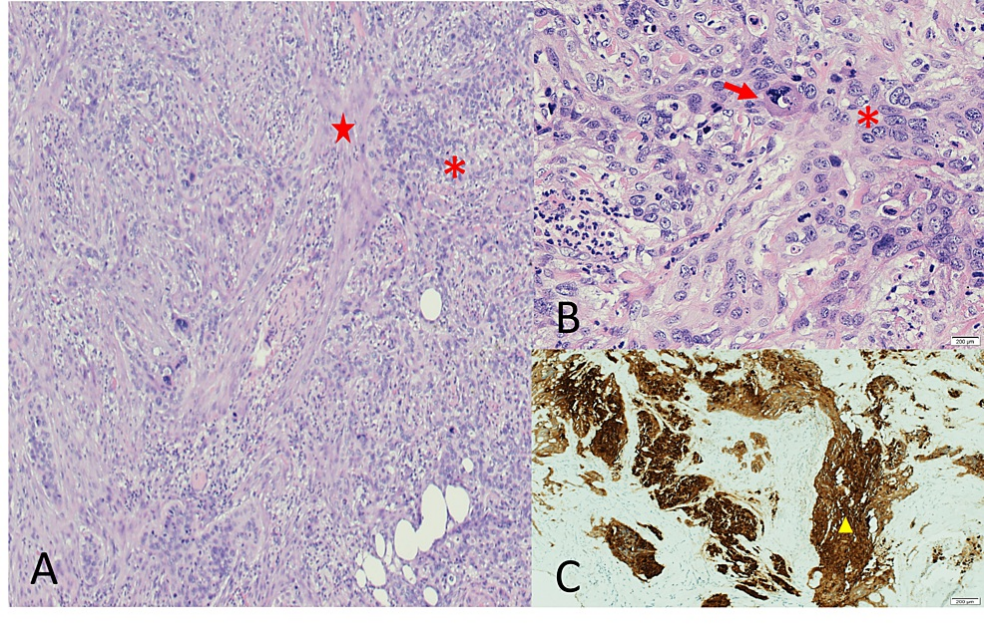
Histopathological examination (HPE) of the endoscopic biopsy specimen with cytokeratin (CK) 5,6 staining. (A) HPE x100 magnification showed the presence of malignant cell islets (asterisk), which are separated by the desmoplastic reaction (star). (B) Further magnified HPE showed the presence of mitotic cells (arrow) and malignant cells with nuclear pleomorphism and hyperchromia (asterisk). (C) Special immunohistochemical staining with CK 5,6 showed a positive (triangle) for malignant cells.

In the interest of imaging findings on lesion location, an endoscopic biopsy of the mass was attempted, which showed malignant pleomorphic cells in nests and singly distributed with desmoplastic reaction (Figure 3) highlighted with cytokeratin (CK) 5,6 immunohistochemical stain (Figure 3C), representing recurrent SCC. Within two weeks, the patient has axial proptosis, a frozen left eye with elevated intraocular pressure, and retropulsion resistant. Owning to the confined mass not extending extraconally, she was then scheduled for urgent modified enucleation with intraconal mass excised that showed SCC with a negative margin in the periorbita region. 

**Figure 4:**
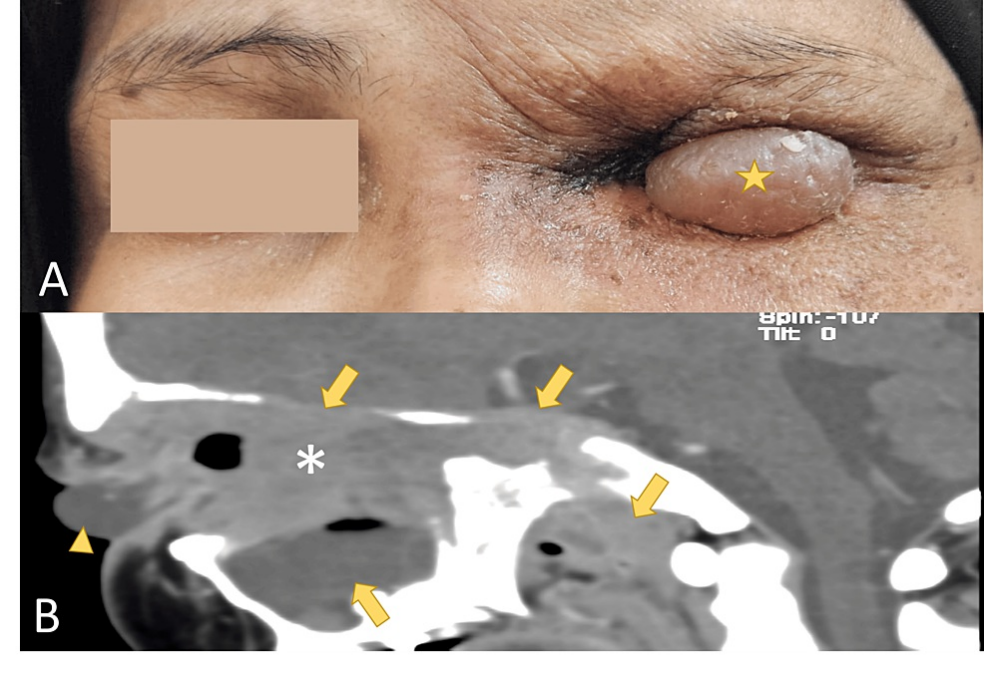
Recurrence after modified enucleation. Recurrence of the non-cutaneous squamous cell carcinoma (ncSCC) over the left eye. (A) Well-defined cystic mass that involves the upper eyelid (star). (B) The sagittal view of contrasted computed tomography orbit showed enhancing extensive tumor (asterisk) occupying orbit and invading maxillary sinus, cavernous sinus, and medial cranial fossa with bony erosion (arrow). A cystic lesion (arrowhead) was arised from the tumor anteriorly that coincides clinically.

After four months, she came with a painful left eye and a non-inflamed cystic mass occupying the orbit (Figure 4A). Urgent computed tomography showed a heterogeneous enhancing lesion in the left orbit extending to the cavernous sinus, middle cranial fossa, left paranasal sinus, and left infratemporal fossa with bony destruction, as in Figure 4B. Tumor debulking was attempted promptly, revealing a hemorrhagic cystic mass with a stalk extending into the depths of the orbit posteriorly and involving the lid margin superiorly, which played out as recurrent SCC. The patient underwent counseling and was followed with palliative care in view of intracranial extension and was not keen on further surgical or medical intervention.

## Discussion

Presentation-wise, lsSCC might be confused with chronic dacryocystitis, namely medial canthal swelling with tearing, in addition to hemolacria [2-4]. Nevertheless, orbital SCC, in contrast, will have frontal pain and numbness with visual impairment and an extraocular motility defect, which might suggest the possibility of perineural infiltration in orbital SCC [5]. The most common site of orbital SCC was the orbital apex, followed by the superomedial orbit and intraconal [1]. In our case, the patient was having typical orbital SCC presentation despite a history of lacrimal SCC while showing features of malignancy aggressiveness over a short period. 

Noteworthy in this case, imaging not only aids in identifying the location of lesions and nature but also helps in deciding the approach of operation. As in this patient, we decided on a nasal approach in the biopsy of the lesion due to its close relation to the medial wall and lies in the posterior orbit that is difficult to approach via anterior orbitotomy. It was effective and minimally invasion with experienced rhinologists. Numerous studies have also shown a considerably high efficiency of 86% to 94%, with transient minor complications like sinusitis, ecchymosis, eyelid edema, and diplopia [6-8]. It was shown that it can reduce proptosis by 1 mm (range -4 to +3) [6].

In terms of treatment, studies suggested radiotherapy for lsSCC after surgical excision due to the high recurrence rate and better prognosis [2-4]. However, radiotherapy and chemotherapy did not associate with a better prognosis from the study for ncSCC. Surgery was the mainstay in prolonging survival rates [1]. After all, there is no standard guideline for ncSCC, which may be due to its rarity and poor prognosis, necessitating further study to determine the benefits and timing of surgery, chemotherapy, and radiotherapy [2,3,5]. With the knowledge of past lsSCC in the patient, the patient had a recurrence despite appropriate initial treatment that ended in relentless tumor recurrence, reminding us of watchful monitoring for the patient. 

## Conclusions

ncSCC is a rare and challenging malignant disease to attend to, especially lsSCC, which carries a high recurrence rate and is likely aggressive in the disease course. In medial wall and intraconal lesions, an endoscopic approach can be considered with a high success rate and acceptable transient complications. Besides surgery, it is important for multidisciplinary care for such patients for further adjuvant therapy, pain management, and palliative care. More studies and guidelines are advised to provide better healthcare for such patients.
